# Quantitative assessment of dry mouth in scrub typhus using salivary scintigraphy

**DOI:** 10.1038/s41598-021-03185-z

**Published:** 2021-12-08

**Authors:** Joo-Hee Hwang, Yeon-Hee Han, MD Tazikur Rahman, Chang-Seop Lee

**Affiliations:** 1 Department of Internal Medicine, Jeonbuk National University Medical School and Hospital, Jeonju, 54896 Republic of Korea; 2 Department of Nuclear Medicine, Jeonbuk National University Medical School, Jeonju, 54896 Republic of Korea; 3 Cyclotron Research Center, Molecular Imaging and Therapeutic Medicine Research Center, Jeonbuk National University Medical School and Hospital, Jeonju, 54907 Republic of Korea; 4 Department of Medical Science, Jeonbuk National University Medical School, Jeonju, Republic of Korea; 5 Research Institute of Clinical Medicine of Jeonbuk National University-Biomedical Research Institute of Jeonbuk National University Hospital, Jeonju, 54907 Republic of Korea

**Keywords:** Diseases, Signs and symptoms

## Abstract

Scrub typhus is an acute febrile illness caused by the intracellular pathogen *Orientia tsutsugamushi*. The clinical features include fever, myalgia, lymphadenopathy, and dry mouth. However, no studies have assessed the symptom of dry mouth in patients with scrub typhus. We investigated the pattern of salivary scintigraphy during the acute febrile state and compared it with any changes after treatment. Fourteen patients underwent both pre- and post-treatment salivary scintigraphy. Imaging analysis was conducted using radioactivity in the oral cavity, parotid glands, and submandibular glands. During the acute phase, the radioactivity in the oral cavity markedly decreased, while that in the parotid and submandibular glands was preserved. After treatment, radioactivity in the oral cavity showed a significant increase at 20-min, 40-min, and after wash-out. The ejection fraction (%) of the parotid glands also increased after treatment. In contrast, the radioactivity levels of the parotid and submandibular glands were not statistically different after treatment. Salivary scintigraphy indicated that insufficient saliva excretion from the salivary glands into the oral cavity was one reason for the dry mouth reported by patients with scrub typhus. In the future, salivary scintigraphy imaging could contribute to the evaluation of dry mouth in patients with scrub typhus.

## Introduction

Scrub typhus is an acute febrile illness caused by the intracellular pathogen *Orientia tsutsugamushi*, which is transmitted to human by an infected chigger bite^1^. Scrub typhus originally was associated with the 'tsutsugamushi triangle' in the Asia–Pacific region, until recent evidence from the Middle East, South America, and Africa suggested a wider global distribution in tropical and subtropical regions^2–5^. The clinical course of scrub typhus varies from self-limiting to fatal illness, with an estimated mortality of 6.0% (median, range 0–70.0%)^6^.

The diagnosis of scrub typhus is based on a history of exposure, clinical features, and results of serologic testing^7^. However, the clinical features of scrub typhus can be similar to those of other infectious diseases, including fever, chills, myalgia, and lymphadenopathy; as a result, diagnostic difficulties often lead to misdiagnoses and contribute to a substantial delay in the correct diagnosis. Dry mouth is one of the most common yet distinctive features reported among scrub typhus patients.

Technetium-99m (Tc-99m) pertechnetate salivary scintigraphy is an imaging modality that shows a series of physiological processes by which saliva is produced and secreted^8^. Salivary scintigraphy helps us to evaluate the function of major salivary glands and the patency of salivary ducts and allows the diagnosis of acute/chronic salivary gland disorders and tumors^9^. It has been performed mostly in patients with radiation-induced xerostomia and autoimmune diseases such as Sjögren syndrome. In these diseases, a decrease in ejection fraction of varying degrees from 10% to more than 30% has been reported. In some extreme cases, the radioactivity uptake is so low that it is almost impossible to measure the ejection fraction^10–12^.

Acute sialadenitis is an infectious or inflammatory disorder of the salivary glands and is caused by bacterial infection, viral infection, non-infectious inflammatory processes such as sarcoidosis, and immune-mediated processes like Sjögren syndrome^13^. Immunofluorescence had revealed that *O. tsutsugamushi* is present in more than 50% of the salivary glands of infected trombidium mites^14^. Humans acquire the pathogen when bitten by trombiculid mite larvae (chiggers) whose salivary gland cells are infected with *O. tsutsugamushi*^15^. Therefore, we hypothesized that, if humans are infected with *O. tsutsugamushi*, then the *O. tsutsugamushi* infection will be present in human salivary glands as well and can lead to a dry mouth. We attempted to prove this theory through salivary scintigraphy during the acute febrile state of scrub typhus and compared the changes before and after appropriate antibiotic treatment.

## Results

### Patients and clinical data

Over the study period, 16 scrub typhus patients were enrolled. Among them, two patients whose follow-up salivary scintigraphy could not be performed due to loss to follow-up were excluded, which left a final total of 14 patients to be included in this study. The median age of the patients was 65.5 years (interquartile range: 55.0–71.0), and eight (57.1%) were female. Seven (50.0%) patients were employed in agricultural activities. The majority of patients presented with a skin rash (9, 64.3%) and eschar (13, 92.9%), and all patients complained of dry mouth. The laboratory findings revealed mild to moderate elevation of liver function tests in most patients despite lack of underlying liver diseases, and about half of the patients had thrombocytopenia on admission. However, the abnormal laboratory findings had normalized by the second visit. In addition, the most commonly identified genotype of *O. tsutsugamushi* was the Boryong strain (13, 92.9%), which is the predominant strain found throughout South Korea. All patients were successfully treated with a 7-day course of oral doxycycline at 200 mg/day. The demographic and clinical characteristics of the enrolled patients are summarized in Table 1.

**Table 1:** The demographic and clinical characteristics and laboratory findings of scrub typhus patients.

Characteristics	Scrub typhus (n = 14)
**Demographic data, median (IQR)**	
Age (years)	65.5 (55.0–71.0)
Female, no. (%)	8 (57.1)
Agricultural activities, no. (%)	7 (50.0)
Duration of illness before admission (days)	5.5 (3.0–9.0)
Hospitalization days	6 (5.0–8.0)
**Comorbidities, no. (%)**	
Connective tissue disease	0
Diabetes mellitus	1 (7.1)
Solid tumor	3 (21.4)
Leukemia/lymphoma	0
**Clinical signs and symptoms, no. (%)**	
Headache	9 (64.3)
Dyspepsia	3 (21.4)
Nausea/Vomiting	3 (21.4)
Abdominal pain	3 (21.4)
Fever	14 (100.0)
Rash	9 (64.3)
Eschar	13 (92.9)
Dry mouth	14 (100.0)
**Laboratory values, median (IQR)**	
WBC count, × 1,000/mm^3^	8.0 (6.5–9.1)
Platelet count, × 1,000/mm^3^	163.5 (105.0–183.0)
PT, INR	1.1 (1.0–1.2)
Total bilirubin, mg/dL	0.64 (0.48–0.98)
Albumin, g/dL	3.8 (3.4–4.0)
AST, IU/L	68.5 (46.0–90.0)
ALT, IU/L	59.0 (38.0–110.0)
Creatinine, mg/dL	0.8 (0.7–0.9)
hs-CRP, mg/dL	62.7 (45.9–76.6)
**Genotype, no. (%)**	
Boryong Strain	13 (92.9)
Unknown	1 (7.1)
**Treatment, no. (%)**	
Doxycycline	14 (100.0)

*IQR* interquartile range, *WBC* white blood cell, *PT* prothrombin time, *AST* aspartate aminotransferase, *ALT* alanine aminotransferase, *ALP* alkaline phosphatase, *CRP* C-reactive protein.

### Qualitative results of salivary scintigraphy

**Figure 1:**
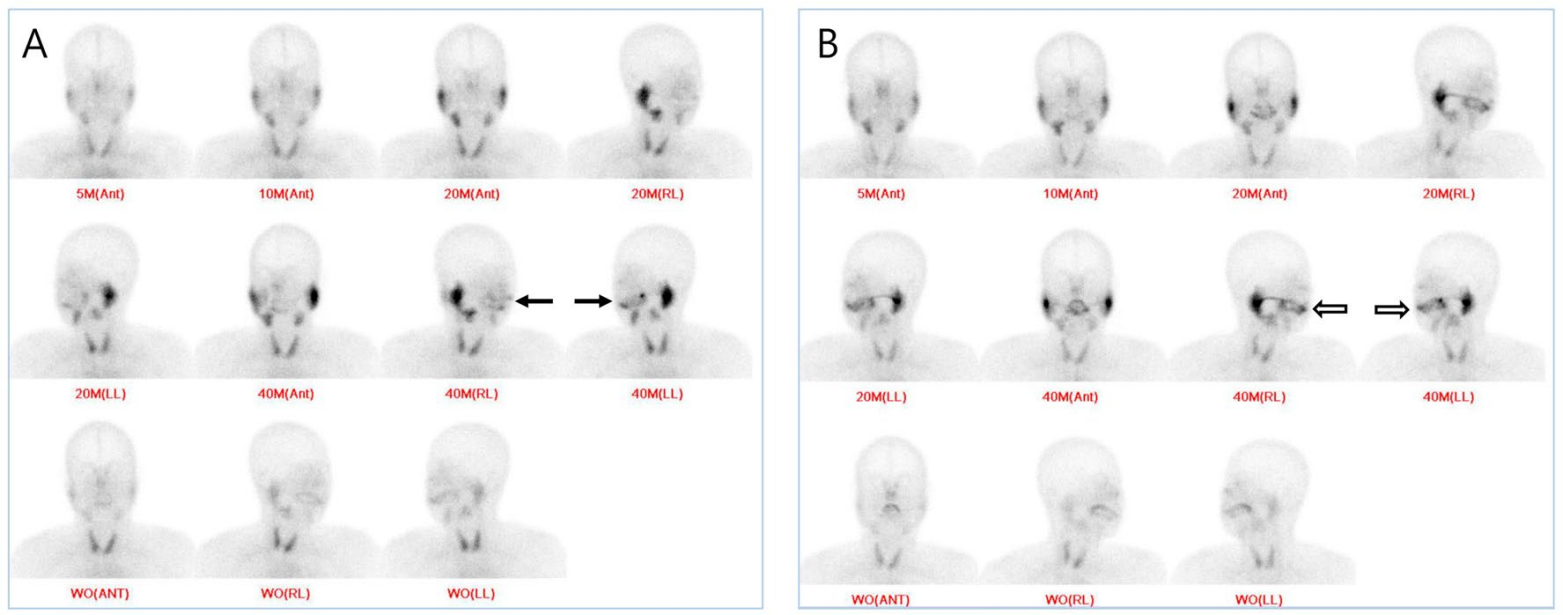
(**A**) Compared to pre-treatment imaging, saliva excretion into the oral cavity markedly decreased at 40 min after technetium-99 m pertechnetate injection (arrow). (**B**) After proper antibiotic treatment, the radioactivity levels in the oral cavity improved to the normal range (open arrow). *Ant* anterior view, *RL* right lateral view, *LL* left lateral view.

The dynamic perfusion images of 14 patients demonstrated that the perfusion pattern was within normal limits both before and after treatment. At the early time points of 5- and 10-min, the radioactivity values in the oral cavity, parotid glands, and submandibular glands showed no significant difference compared to the result before and after treatment. In contrast, the radioactivity in the oral cavity at 20-min and 40-min had markedly decreased on pre-treatment salivary scintigraphy and recovered on post-treatment scintigraphy (Fig. 1A,B).

### Quantitative results of salivary scintigraphy

**Figure 2:**
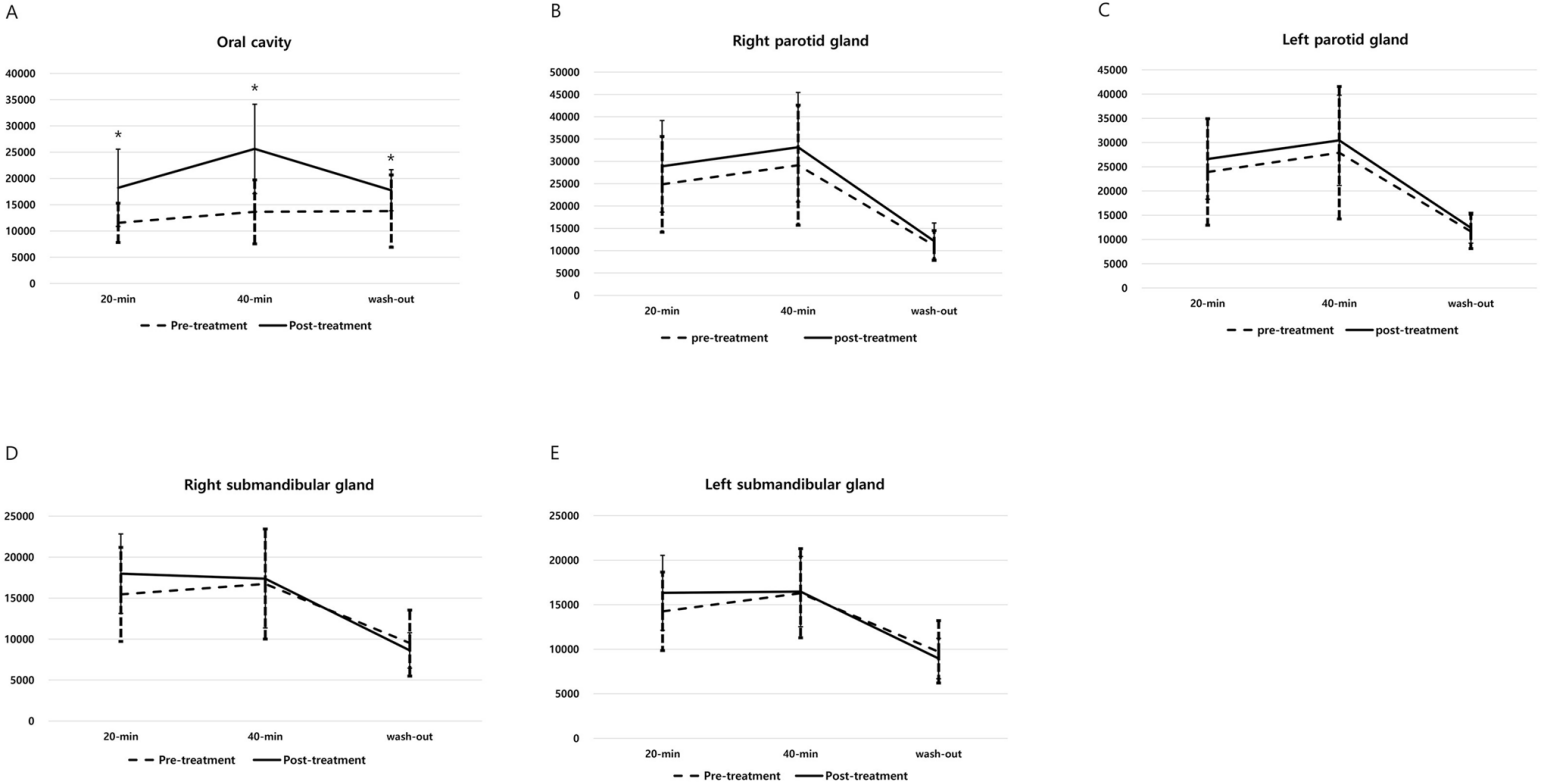
(**A**) The radioactivity levels in the region of interest (ROI) of the oral cavity showed a significant increase after proper treatment at all time points. An asterisk (*) indicates statistical significance. (**B**,**C**) The radioactivity values in bilateral parotid glands were increased slightly following treatment; however, the differences were not statistically significant. (**D**,**E**) Although there was no statistical significance, similar to the radioactivity in the parotid glands, the radioactivity of the submandibular glands slightly increased at 20-min and 40-min after treatment.

After proper antibiotic treatment, the ejection fraction (%) of the right parotid gland statistically increased from 53.86 ± 7.00 (%) to 61.21 ± 9.78 (%), while that of the left parotid gland increased from 53.36 ± 8.67 (%) to 58.36 ± 10.64 (%). *P*-values were 0.009 and 0.030, respectively. In general, an ejection fraction greater than 50% is considered normal^16^. When analyzing strictly, it is recommended that the ejection fraction should be used after confirming the normal reference for each hospital equipment. In the hospital of this study, 55% has been used as a normal cutoff value. The radioactivity in the region of interest (ROI) of the oral cavity showed a statistically significant increase after antibiotic treatment at 20-min, 40-min, and after wash-out (Fig. 2A). Although the radioactivity values in the parotid glands increased slightly at 20-min, 40-min, and after wash-out following appropriate treatment, the differences were not statistically significant. In addition, the radioactivity gaps between the pre-and post-treatment levels on wash-out images were smaller than those on the 20-min and 40-min images (Fig. 2B,C). Similar to the radioactivity in the parotid glands, that of the submandibular glands showed a slight increase on the 20-min and 40-min images after treatment. The radioactivity gaps between pre-and post-treatment gradually decreased on the images from 20-min to 40-min. Then, the radioactivity on the wash-out images after treatment was lower than the values before treatment, suggesting a better response to the sialagogue (Fig. 2D,E). The radioactivity results in the ROIs of the oral cavity, parotid glands, and submandibular glands both before and after treatment are summarized in Table 2.

**Table 2:** Radioactivity in the regions of interest of the oral cavity, parotid glands, and submandibular glands of patients before and after treatment.

Site		20-min	40-min	Wash-out
Oral cavity	Before	11,551.06 ± 3749.33	13,636.78 ± 6099.56	13,800.08 ± 6889.79
	After	18,216.40 ± 7354.61	25,630.24 ± 8510.61	17,754.11 ± 3902.61
	P-value	0.001	0.001	0.041
Right parotid gland	Before	24,865.53 ± 10,727.57	29,148.01 ± 13,433.72	11,152.12 ± 3340.39
	After	28,902.56 ± 10,267.79	33,186.32 ± 12,246.14	12,145.07 ± 4052.25
	P-value	0.124	0.140	0.551
Left parotid gland	Before	23,930.24 ± 10,993.46	27,911.54 ± 13,662.23	11,659.42 ± 3547.76
	After	26,606.08 ± 8287.87	30,449.12 ± 9308.72	12,418.93 ± 3177.33
	P-value	0.109	0.300	0.510
Right submandibular gland	Before	15,454.47 ± 5736.58	6726.21 ± 6713.08	9514.88 ± 4024.44
	After	17,985.42 ± 4849.34	17,367.57 ± 6005.85	8618.90 ± 2155.84
	P-value	0.221	0.778	0.510
Left submandibular gland	Before	14,258.63 ± 4401.66	16,290.93 ± 5007.00	9712.44 ± 3503.94
	After	16,333.09 ± 4207.31	16,473.84 ± 3939.02	8952.81 ± 2265.00
	P-value	0.158	0.975	0.510

## Discussion

Dry mouth is one of the most common clinical features reported by patients with scrub typhus. In this study, we demonstrated using salivary scintigraphy that dry mouth is caused by decreased excretory capacity from the salivary glands in the oral cavity. We investigated the pattern of salivary scintigraphy in the acute phase of scrub typhus and compared the changes in radioactivity before and after appropriate treatment over a two years period.

A previous study reported that *O. tsutsugamushi* is intermingled with secretory granules in the cytoplasm of salivary gland cells in infected trombiculid larvae^15^. Similarly, *O. tsutsugamushi* likely affects the salivary glands in patients with scrub typhus, although the responsible pathophysiology is not understood.

Tc-99 m pertechnetate salivary scintigraphy is a non-invasive, reliable, and widely accepted imaging modality that has been used broadly to assess major salivary gland function, particularly in the parotid and submandibular glands. The salivary epithelial cells express a number of sodium/iodide symporters (NIS), which take up univalent anions such as I^−^ and Cl^−^^17^. Tc-99 m pertechnetate (^99m^TcO4^-^) is taken up by salivary epithelial cells through NIS and is secreted into saliva^17^. After accumulation of ^99m^TcO4^-^ in the salivary glands, loading of sialagogues, such as sour juice or vitamin C powder, stimulates the secretion of saliva, which enables an evaluation of the excretory function of salivary glands and any obstruction of the salivary ducts.

Salivary scintigraphy has been used widely to assess multiple salivary diseases, primarily radiation-induced xerostomia and autoimmune diseases like Sjögren syndrome. A previous study that examined radiation-induced injury of the salivary glands reported that xerostomia was based mainly on the failure of the glands to excrete saliva in the early period following high-dose irradiation, in contrast, decreased uptake ability played a significant role in the later period^18^. Other studies investigating patients with Sjögren syndrome have demonstrated delayed uptake, reduced concentration, and/or delayed excretion of radiotracer in the salivary scintigraphy^19–21^. As previous studies have shown, salivary scintigraphy in these diseases is characterized by a markedly decreased uptake ability. In the present study, however, we observed relatively preserved ability of the parotid and submandibular glands in patients with scrub typhus to take up the radiotracer. Markedly decreased excretory function was noted in the acute phase of scrub typhus. The decreased excretory ability of the glands fully recovered after appropriate management in all patients.

We also found that the radioactivity of the oral cavity showed a statistically significant increase after treatment, while the radioactivity of parotid and submandibular glands slightly increased without statistical significance. These ironic results suggest the possibility that the minor salivary glands, which are widely distributed in the mucosa of cheek, lip, tongue, soft palate, and oral floor, could also be involved to a non-negligible level.

We are aware of a few limitations of this study. First, it was a single-center study that included a small number of patients, so the results should be interpreted with caution and should not be generalized. Nevertheless, the salivary scintigraphy findings of all patients in this study demonstrated similar findings, and we believe that the results would be the same in other scrub typhus patients. In addition, we could not prove sialadenitis pathologically due to the invasiveness of obtaining salivary gland tissue from patients. An additional limitation was that it was not possible to separate the radioactivity of the oral cavity from that of the sublingual glands. Because the sublingual gland is the smallest among the major salivary glands and accounts for only about 10% of saliva production^22^, it is believed that oral cavity radioactivity is a reliable measure. This inevitable shortcoming, which is present when using two-dimensional image analysis, could be overcome by obtaining and analyzing single-photon emission computed tomography images. Despite these limitations, we objectively evaluated the symptom of dry mouth in patients with scrub typhus using quantitative values of salivary scintigraphy.

To our knowledge, this study is the largest study to assess salivary scintigraphy in patients with scrub typhus and to conduct a quantitative analysis. We observed that the radioactivity of the oral cavity significantly increased after appropriate treatment. We also showed that the radioactivity of the parotid and submandibular glands was preserved during the acute phase of scrub typhus. Based on these findings, we concluded that insufficient saliva excretion from the salivary glands into the oral cavity is one reason for the dry mouth reported by patients with scrub typhus. As salivary scintigraphy provides an objective measure of the physiologic status of saliva production with quantitative values, it could contribute to the evaluation of dry mouth in patients with scrub typhus.

## Methods

### Patients and data collection

Sixteen eligible scrub typhus patients (≥ 18 years of age) were recruited prospectively upon admission to a 1200-bed tertiary hospital in South Korea between October 2018 and December 2020. Pre-treatment Tc-99 m pertechnetate salivary scintigraphy was performed within 12 h of hospital admission, and post-treatment salivary scintigraphy was acquired three weeks after completion of appropriate treatment. Laboratory diagnosis of scrub typhus was made according to one of the following criteria: (1) increase in indirect immunofluorescence assay (IFA) IgM titer ≥ 1:160 against *O. tsutsugamushi*; (2) increase in IFA IgG titer ≥ 1:256; (3) ≥ fourfold increase in IFA titer in paired sera; and (4) positive result from nested polymerase chain reaction (PCR) targeting the 56-kDa gene of *O. tsutsugamushi*. Demographic and clinical information was collected retrospectively from electronic medical records and included age, sex, comorbidities, clinical information, and laboratory values.

### Genotyping by DNA amplification and sequencing

Peripheral blood mononuclear cells obtained from acute-phase blood samples of scrub typhus patients were purified using a QIAamp DNA Blood Mini Kit (QIAGEN GmbH, Hilden, Germany) according to the manufacturer's protocol. Nested PCR for the 56-kDa gene of *O. tsutsugamushi* was performed as described previously by Kim et al.^23^. The amplified PCR products were confirmed by 1.2% agarose gel electrophoresis, purified using a QIAquick gel extraction kit (QIAGEN), and sent to COSMO Genetech (Seoul, Korea) for sequencing.

### Imaging protocol of salivary scintigraphy

All patients fasted for at least 4 h before the injection of radiopharmaceutical. Scanning began at the time of the intravenous injection of 555 MBq of Tc-99 m pertechnetate (^99m^TcO4^-^) using a gamma camera (Symbia Intevo 16, Siemens Medical Solutions, Munich, Germany, 2016) with an energy peak setting of 140 keV ± 7.5% for Tc-99 m. Twenty dynamic perfusion images were obtained for 3 s per image. Static images were collected at 5-min, 10-min, 20-min, and 40-min after injection and acquired for 90 s per image. At 5-min and 10-min, only anterior images were obtained, while at 20-min and 40-min, right lateral and left lateral images were obtained as well (Fig. 1). After acquiring the 40-min images, the patients drank 200 mL of orange juice as a sialagogue. Then, anterior and bilateral lateral images labeled 'wash-out images' were acquired to evaluate the salivary gland response to sialagogue. All patients underwent pre-treatment and post-treatment salivary scintigraphy.

### Qualitative and quantitative analyses of salivary scintigraphy

**Figure 3:**
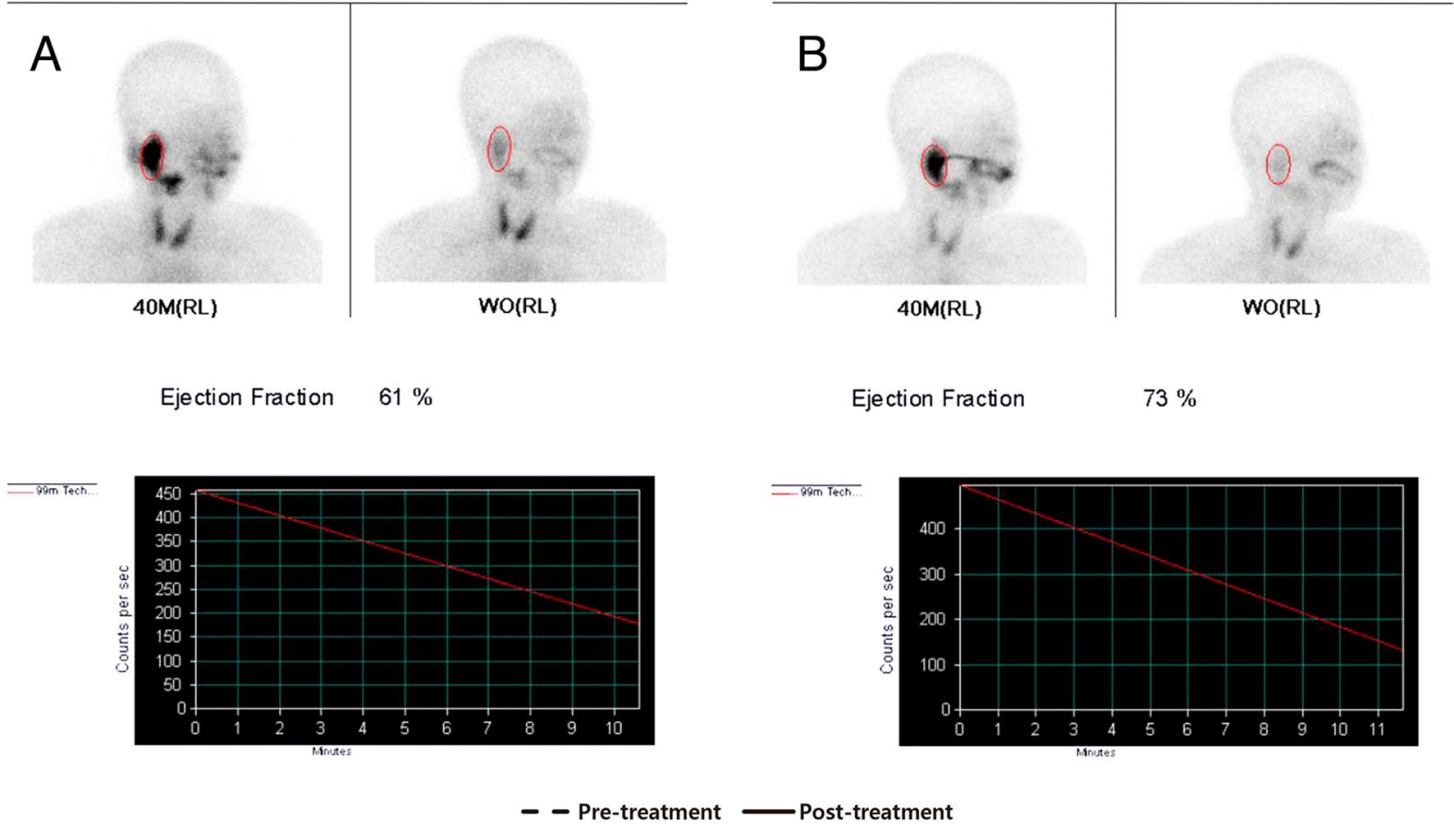
The ejection fraction (%) of the right parotid gland was calculated by comparing the radioactivity level before and after administration of sialagogue (orange juice, 200 mL). The ejection fraction increased from 61% (**A**) to 73% (**B**) after antibiotic treatment. *RL* right lateral view.

**Figure 4:**
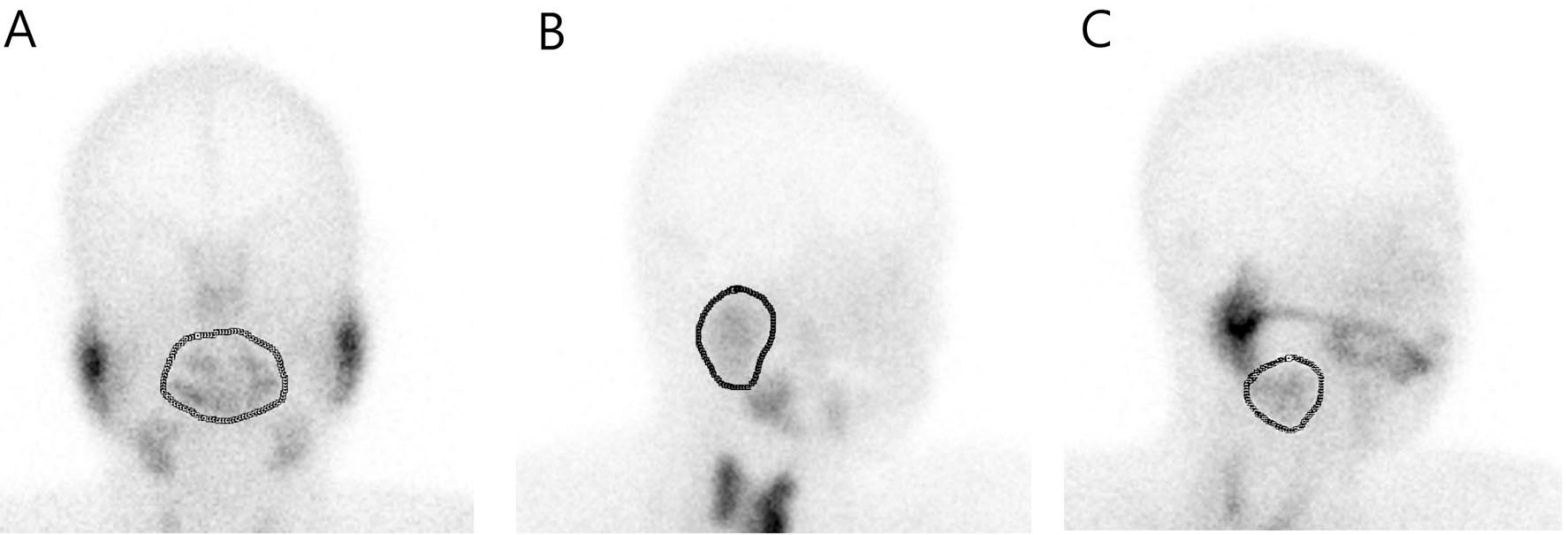
(**A**) The ROI of the oral cavity was drawn manually along the outline of the radioactivity on the anterior view. (**B**,**C**) The ROIs of the parotid and submandibular glands were drawn along the outline of the organs on the lateral views. These radioactivity information was measured using PMOD software (version 3.7; PMOD Technologies LLC, Zurich, Switzerland, https://www.pmod.com/web/).

Qualitative analysis of Tc-99 m pertechnetate salivary scintigraphy was performed on a picture archiving and communication system (PACS M6, INFINITT Health care, Seoul, South Korea), and quantitative analysis was carried out using PMOD software (version 3.7; PMOD Technologies LLC, Zurich, Switzerland, https://www.pmod.com/web/) by drawing ROI in the oral cavity, parotid glands, and submandibular glands. The difference (%) between radioactivity counts in the ROI of the parotid glands on the lateral view of the 40-min and wash-out images was termed the ejection fraction (%) (Fig. 3). The radioactivity of the oral cavity was measured by drawing ROIs on the anterior views at 20-min, 40-min, and also on the wash-out images (Fig. 4A). The ROIs of the parotid and submandibular glands were drawn along the outlines of the organs on the lateral views of the 20-min, 40-min, and wash-out images (Fig. 4B,C).

### Statistical analysis

Descriptive statistics were expressed as numbers and percentages, means and ranges, or medians and interquartile ranges (IQRs). Statistical analyses were performed with the Wilcoxon signed-rank test using MedCalc for Windows, version 20 (MedCalc Software, Mariakerke, Belgium, https://www.medcalc.org/). In all analyses, a two-tailed *P*-value < 0.05 was considered statistically significant.

### Ethical statement

This study was approved by the Institutional Review Board (IRB) of Jeonbuk National University Hospital, and all patients provided written informed consent (IRB registration number 2019-05-044-005). This study was also conducted in accordance with Good Clinical Practice Guidelines and the Declaration of Helsinki.
